# Early Retinal Microvascular Changes Assessed with Swept-Source OCT Angiography in Type 1 Diabetes Patients without Retinopathy

**DOI:** 10.3390/jcm12072687

**Published:** 2023-04-04

**Authors:** Pétra Eid, Catherine Creuzot-Garcher, Ludwig Serge Aho, Pierre-Henry Gabrielle, Estelle Charpin, Déa Haddad, Laure-Anne Steinberg, Alain Bron, Bruno Verges, Louis Arnould

**Affiliations:** 1 Ophthalmology Department, University Hospital, 21000 Dijon, France; 2 Centre des Sciences du Gout et de l’Alimentation, AgroSup Dijon, CNRS, INRAE, University of Burgundy Franche-Comté, 21000 Dijon, France; 3 Epidemiology Department, University Hospital, 21000 Dijon, France; 4 Endocrinology-Diabetology Department, University Hospital, 21000 Dijon, France; 5 INSERM, LNC-UMR1231, 21000 Dijon, France; 6 INSERM, CIC1432, Clinical Epidemiology Unit, Dijon University Hospital, 21000 Dijon, France

**Keywords:** swept-source optical coherence tomography angiography, type 1 diabetes, FreeStyle Libre, retinal vascular densities, superficial capillary plexus, deep capillary plexus

## Abstract

Type 1 diabetes is a chronic disease that can lead to vision loss when diabetic retinopathy develops. Retinal microvascular alterations occur before the appearance of clinical signs on a fundus examination. This study aimed to analyze retinal vascular parameters on optical coherence tomography angiography (OCT-A) in patients with type 1 diabetes without diabetic retinopathy in comparison with non-diabetic volunteers. This cross-sectional study was conducted at Dijon University Hospital from 2018 to 2020. Vascular densities were measured using macular OCT-A. In total, 98 diabetes patients and 71 non-diabetic volunteers were enrolled. A statistically significant lower vascular density of the inner circle was found in the superficial capillary plexus (SCP) in the diabetes group (*p* < 0.01). There was a statistically significant correlation between central vascular density in the deep capillary plexus (DCP) and total daily insulin intake (*p* = 0.042); furthermore, use of the FreeStyle Libre (FSL) device was associated with higher vascular densities in both the SCP (*p* = 0.034 for outer circle density) and DCP (*p* < 0.01 for inner circle density and *p* = 0.023 for outer circle density). Retinal microvascularization was early-altered in type 1 diabetes, and using the FSL device seemed to preserve retinal microvascularization.

## 1. Introduction

Type 1 diabetes is among the most common chronic diseases globally impacting young people [1]. This metabolic disorder affects insulin-producing β cells in the pancreas, leading to a profound lack of insulin and subsequent hyperglycemia [2]. Retinopathy is one of the most harmful microvascular complications of type 1 diabetes. Early screening is, therefore, essential to avoid proliferative retinopathy and blindness. This screening is based on fundus modifications, classifying the retinopathy from minor non-proliferative retinopathy to eye-threatening proliferative retinopathy [3]. Vascular modification caused by chronic exposure to abnormal high glucose levels is key in the development of diabetic retinopathy [4]. Retinal angiography with a fluorescein dye injection remains the gold standard for evaluating vascular alterations in diabetic retinopathy by enabling the direct visualization of the microvasculature. However, fluorescein angiography is invasive and vascular modifications are most often evidenced at advanced stages, which already constitute a sight-threatening condition.

The retina is human tissue in which a direct in vivo visualization of the vascular network is possible. The emergence of novel technologies has provided new insights into the pathophysiology of diabetic retinopathy along with many new questions and hypotheses to be explored. Swept-source optical coherence tomography angiography (SS OCT-A) is a recent retinal imaging technology that enables the three-dimensional visualization and quantitative analysis of retinal microvascularization and the foveal avascular zone (FAZ). The software associated with the machine facilitates the quantitative analysis of the superficial and deep retinal capillary plexuses (SCP and DCP, respectively) [5]. This retinal imaging technique represents a non-invasive, accurate, quantitative, and reproducible approach for the evaluation of retinal microvascular changes.

The development of microvascular lesions in diabetes starts with biochemical changes, leading to functional and then anatomical vascular changes. The appearance of anatomical lesions occurs after years of retinal exposure to chronic hyperglycemia, and includes pericyte loss, capillary rarefaction, microaneurysms, intraretinal microvascular abnormalities, and ischemic injury [6]. The most well-known risk factors of diabetic retinopathy are glycemic control and the duration of diabetes, but these determinants do not fully explain an individual’s risk of diabetic retinopathy [7,8]. Indeed, various studies have suggested other multiple and modifiable risk factors of microvascular injury in the development and progression of clinical diabetic retinopathy such as hypertension, smoking, dyslipidemia, and obesity [9].

Various studies have focused on microvascular modifications during diabetic retinopathy, but only a few have examined early microvascular changes before the onset of diabetic retinopathy in type 1 diabetes, reporting heterogeneous results [10,11,12]. Although several studies have found modifications of several OCT-A parameters such as FAZ enlargement and perfusion density, the correlation with glycated hemoglobin (HbA1c), diabetes duration, or other risk factors is poorly documented and discussed, particularly in type 1 diabetes [10,11]. Most studies on OCT-A and diabetes were performed using 3 × 3 mm angiograms from spectral-domain OCT-A, restraining the analysis to a limited area surrounding the fovea. In this study, we aimed to investigate microvascular changes with SS OCT-A using 6 × 6 mm angiograms. This wider acquisition could be more informative of potential retinal changes outside the 3 mm surrounding the fovea. Furthermore, more precise images can be acquired with swept-source technology, thereby facilitating a better analysis of the DCP.

This study was based on the hypothesis that early retinal microvascular changes can be identified in type 1 diabetic patients without diabetic retinopathy, and that the retinal microvascular changes are correlated with glycemic control and diabetes monitoring.

We aimed to: (a) compare SS OCT-A data between patients with type 1 diabetes without diabetic retinopathy and non-diabetic volunteers; (b) analyze the association between the retinal microvascular quantitative parameters and glycemic control, assessed by the HbA1c level and the MAGE (mean amplitude of glycemic excursion) score; and (c) determine whether diabetes management with continuous glucose monitoring had an impact on the OCT-A parameters.

## 2. Materials and Methods

### 2.1. Participants

We conducted a cross-sectional observational study in the Endocrinology and Ophthalmology Departments of Dijon University Hospital from February 2018 to February 2020.

Patients with type 1 diabetes without diabetic retinopathy constituted the diabetes group and non-diabetic participants were the control group.

All participants underwent an ophthalmological examination, including non-mydriatic color fundus photography and SS OCT-A imaging. Demographic, medical history, clinical examination, and laboratory data were collected from endocrinology medical records for the diabetes group. Diabetes patients constituting the diabetes group were recruited in the endocrinology department during a follow-up in consultation or during hospitalization. Volunteers constituting the control group were patients consulting the ophthalmology department for various purposes, excluding patients with retinal diseases, diabetic retinopathy, or glaucoma. The exclusion criteria for both groups were patients with a history of retinal disease, glaucoma, or a poor-quality SS OCT-A image (signal strength of ≤7/10, the presence of artifacts due to eye movement, or media opacities). The study adhered to the tenets of the Declaration of Helsinki and followed the STROBE recommendations [13]. Approval from the local ethics committee was obtained (no. 2017-A02724-49), and written informed consent was also obtained for each participant.

### 2.2. Imaging

This study was conducted with 6 × 6 mm macular angiograms acquired with an SS OCT-A (PLEX Elite 9000 Swept-Source, Carl Zeiss Meditec, Inc., Dublin, OH, USA) device. Only images with a signal strength of >7/10 and without significant artifacts were retained. In addition, color fundus photography (Visucam Pro, Carl Zeiss Meditec, Inc., Jena, Germany) analyzed by two experts (L.A. and P.H.G.) was used to assess the absence of maculopathy, diabetic retinopathy, and optic disc neuropathy.

The study of quantitative retinal microvascularization was based on the analysis of vessel density (given in mm^−1^) in both the SCP and DCP. The area size and circularity index of a specific retinal zone devoid of vessels in the SCP, named the “foveal avascular zone” (FAZ size given in mm^2^; the FAZ circularity index was unitless), were also studied. All quantitative SS OCT-A data were obtained using the Macular Density v0.7.3 algorithm available online through the ARI Network (Carl Zeiss Meditec, Inc., Dublin, OH, USA). This algorithm includes the removal of projection artifacts, enabling a more refined analysis of the DCP.

The retinal vascular parameters were analyzed on different macular regions corresponding with the Early Treatment Diabetic Retinopathy Study (ETDRS) distribution, in which the center corresponds with a 1 mm diameter circle in the center of the macula and the inner circle and outer circle correspond with circles of 3 mm and 6 mm in diameter surrounding the macula center, respectively. The inner and outer circle are both divided into four different quadrants: superior, nasal, temporal, and inferior.

### 2.3. Mean Amplitude of Glycemic Excursions (MAGE)

For all patients in the diabetes group equipped with a blood glucose sensor linked to a FreeStyle Libre (FSL) device (Abbott Laboratories, Chicago, IL, USA), we collected blood glucose data, measured continuously to evaluate glycemic variability estimated by the MAGE score. The MAGE was calculated using the mean glycemic excursions exceeding one standard deviation of all blood glucose measures over a 48-h period [14].

### 2.4. Statistical Analysis 

STATA software (StataCorp, version 15.1, LLC, College Station, TX, USA) was used for the statistical analysis. The categorical variables were expressed as numbers (percentage) and the continuous variables as medians (interquartile range) where appropriate. The chi-squared test or Fisher’s exact test, when appropriate, were used to compare the categorical variables. The Mann–Whitney test and a multivariate linear regression were used to compare the quantitative data. The analysis was performed with a robust variance estimator. Linearity was assessed using a fractional polynomial model. A two-tailed *p*-value of <0.05 was considered to be statistically significant. The sample size was calculated from an anticipated mean difference between both groups of 0.79 mm^−1^ and a common standard deviation of 1.5 mm^−1^. Taking 0.05 as the alpha risk and a power of 90%, a sample size of 148 patients (ratio 1:1 between both groups) was considered to be adequate. Given the fact that a good correlation was found between both eyes in terms of the quantitative vascular parameters, we chose to keep one eye for the analysis [15]. The left eye was analyzed for participants born in even-numbered years and the right eye was analyzed for those born in odd-numbered years. When the angiogram was uninterpretable in one eye, the other was retained for the analysis.

## 3. Results

A total of 98 patients with type 1 diabetes without diabetic retinopathy (diabetes group) and 71 volunteers without diabetes (control group) were included in this study.

### 3.1. Characteristics of the Population

The characteristics of the population are described in Table 1. The demographic characteristics were similar between both groups. In the diabetes group, the median time since the diabetes diagnosis was 18.0 (8.75–24.0) years and the median HbA1c level was 7.70 (7.20–8.78)%. The MAGE score was available for 46 patients monitored using an FSL device, with a median MAGE score of 79.5 (63.9–101.0) mg/dL being recorded. Overall, 56% (53 patients) of the diabetic group was equipped with an insulin pump, whereas the rest were treated with a basal/background insulin scheme.

### 3.2. Retinal Microvascularization Comparison between the Diabetes and Control Groups

**Figure 1:**
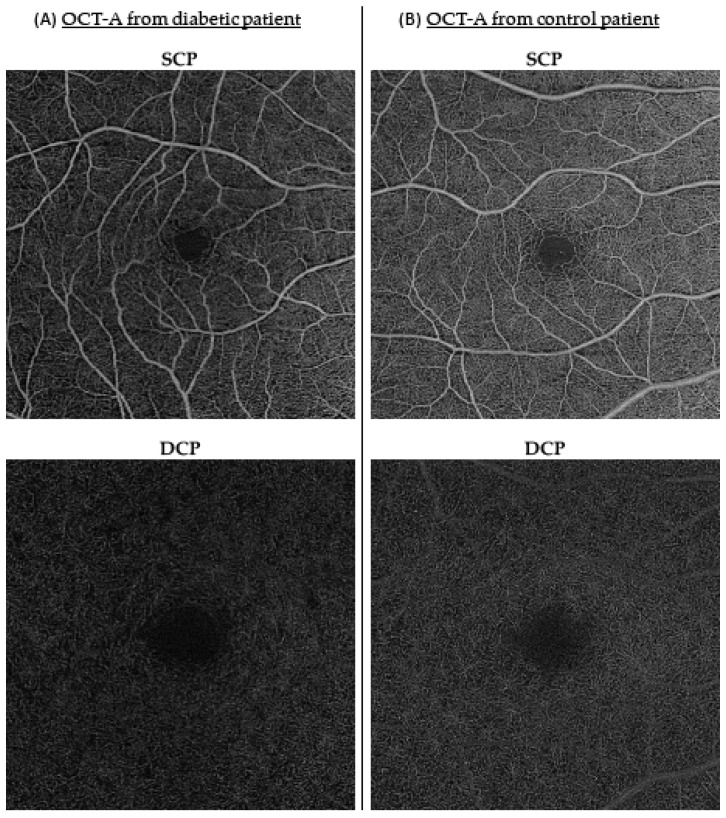
Examples of OCT-A images: illustrations of SCP and DCP in a diabetic patient (**A**) and control patient (**B**). OCT-A: optical coherence tomography angiography; SCP: superficial capillary plexus; DCP: deep capillary plexus.

The inner circle vascular density in the SCP was significantly lower in the diabetes group than in the control group (β = 1.00 (95% CI, 0.468; 1.79), *p* = < 0.01) after adjusting for age and gender (Table 2). The vascular density was also reduced in each segment of the ETDRS grid in the diabetic patients versus controls, although it was not statistically significant for all segments (β = 0.981 (95% CI, 0.475; 1.61), *p* < 0.01 for inner superior density in the SCP; β = 1.08 (95% CI, 0.502; 1.86), *p* < 0.01 for inner nasal density in the SCP; β = 1.06 (95% CI, 0.469; 1.83), *p* < 0.01 for inner inferior density in the SCP; and β = 0.912 (95% CI, 0.136; 1.69), *p* = 0.021 for inner temporal density in the SCP). Those variations are illustrated on Figure 1, with examples of OCT-A images in SCP and DCP obtained from diabetic and control patients. Age appeared to be a confounding factor for various vascular density parameters in both the SCP and DCP (β = −0.0390 (95% CI, −0.0674; −0.00619), *p* < 0.01 for inner circle density in the SCP; β = −0.0510 (95% CI, −0.0784; −0.0284), *p* < 0.001 for inner superior density in the SCP; β = −0.0315 (95% CI, −0.0621; −0.00161), *p* = 0.028 for inner inferior density in the SCP; and β = −0.0570 (95% CI, −0.0990; −0.0150), *p* < 0.01 for outer circle density in the SCP). Vascular density in the outer circle was not statistically different between both groups in this multivariate analysis. No significant changes were found between both groups for vascular densities in the DCP (center, inner circle, and outer circle) or for the FAZ parameters.

### 3.3. Retinal Microvascularization and Diabetic Characteristics in the Diabetes Group

As presented in Table 3, we analyzed the association between the retinal vascular parameters and certain diabetes characteristics linked to the disease course, treatments, and glycemic balance. There was a statistically significant correlation between the total daily insulin intake and central density in the DCP (r = 0.294 (95% CI, 0.00223; 0.539), *p* = 0.042). However, we did not find any statistically significant correlation between the SS OCT-A parameters and diabetes duration, HbA1c, or MAGE score.

Finally, as presented in Table 4, we found a statistically significant association between the use of a technological device such as the FSL for diabetes management and OCT-A vascular parameters in both the SCP (β = 0.922 (95% CI, 0.226; 1.87), *p* = 0.034 for outer circle density) and the DCP (β = 1.92 (95% CI, 0.520; 3.24), *p* < 0.01 for inner circle density and β = 1.80 (95% CI, 0.427; 3.24), *p* = 0.023). No significant association was found for the FAZ parameters.

## 4. Discussion

In this study, we found early retinal microvascular changes in type 1 diabetes without diabetic retinopathy. These modifications were mainly observed in the 3 mm area surrounding the fovea in the SCP. The early microvascular changes in the SCP without rarefaction in the DCP might seem surprising considering that microaneurysms, one of the first signs of diabetic retinopathy, appear more frequently in the inner nuclear layer, corresponding with the DCP in OCT-A studies [16]. Nevertheless, the literature is conflicting. A few studies showed early modification in the SCP [17,18], whereas others found retinal vessel rarefaction predominantly in the DCP [19,20]. The anatomy of the SCP corresponds with the vascularization lying in the nerve fiber layer and ganglion cell layer [21]. Recent studies have suggested that the neurovascular unit, including endothelial cells, pericytes, retinal neurons, and glial cells, is the primary site of early retinal changes, with a thinning of the nerve fiber and ganglion cell layers in type 1 diabetes even before the development of clinical diabetic retinopathy [18]. Another hypothesis is that the DCP is more challenging to analyze because of the deeper localization, thereby resulting in projection artifacts from superficial vessels that could lead to a less precise quantitative analysis. However, our imaging protocol used an algorithm that removed projection artifacts for the DCP analysis in order to limit this phenomenon. We could not ignore the heterogeneity in the vascular plexus localization in OCT-A-based investigations. The SCP and DCP are anatomically and functionally connected; thus, abnormalities in one plexus could lead to modifications in the other [21].

We did not find an early modification in the size or circularity of the FAZ in the diabetes group, similar to previous studies [19,20,22]. The FAZ has been shown to have a high interindividual heterogeneity and a low sensitivity and specificity in detecting diabetic retinopathy compared with other vascular parameters [23,24]. Moreover, it is interesting to note that the FAZ can be explored by various measures, depending on the algorithm used to analyze the OCT-A images. Thus, comparisons are difficult between studies.

We did not find any correlation between the retinal microvascular quantitative parameters and general diabetic control variables such as HbA1c, MAGE, and the onset of diabetes. However, we identified a positive correlation between the total insulin intake and central vascular density in the DCP. Similar results in the literature showed a positive correlation between the total daily insulin intake and ganglion cell complex thickness [25]. A high total daily insulin intake is a surrogate for intensive treatment, linked to a long-term beneficial effect on vascular damage [26,27,28]. Regarding the glycemic control analysis through the HbA1c and MAGE assessment, it is essential to remember that these parameters only reflect the last few days to months of the glucose balance. On the other hand, OCT-A vascular changes represent months to years of anatomical remodeling, which could explain the absence of any correlation with these parameters linked to a short-term glycemic variation. Moreover, our diabetes group comprised patients with regular follow-ups in the endocrinology department. Such a close medical follow-up should lead to a better glycemic balance, but it also enhances the screening and management of other general cardiovascular risk factors. Thus, this selection of patients may have led to a potential selection bias. It would be interesting to compare retinal microvascular parameters between our patients and another group with systemic complications linked to diabetes that require a medical follow-up in hospital. Finally, the median age of our diabetes group was close to 30 years. The risk of microvascular and macrovascular complications linked to diabetes increases with age, but it appears that this risk is particularly low before the age of 20 years [29]. In the present study, the few statistically significant differences found in the OCT-A parameters could be influenced by the young age of our population.

Half of the diabetes patients were equipped with an insulin pump and/or had an FSL device for continuous glucose monitoring. Thanks to advances in glycemic control methods, these types of equipment improve glycemic management and are associated with a decrease in systemic vascular complications [26,27,30]. Interestingly, our investigation found that continuous glucose monitoring using devices such as the FSL was associated with higher retinal vascular densities in the SCP and DCP. One could speculate that this technology could slow down retinal microvascular rarefaction in type 1 diabetes and may delay the appearance of diabetic retinopathy. Moreover, the significant proportion of patients equipped with technological devices enabling the optimization of glycemic management was an interesting and original characteristic of our diabetes population. Indeed, these novel technologies, by promoting a better glycemic balance, could reduce smooth-out OCT-A changes in diabetic patients. Further longitudinal studies are needed to assess whether continuous glucose monitoring using novel devices such as the FSL might help to preserve normal microvascular networks over the long term and reduce the risk of progression to clinical diabetic retinopathy.

We acknowledge a few limitations in our study. First, the impact of other systemic features that could interfere with the vascular density analysis was only partially controlled. Second, as for many other OCT-A studies, we showed that age was a major confounding factor that must be considered when interpreting retinal microvascular changes [24]. Both groups showed similar demographic characteristics, limiting the impact of age on the OCT-A comparison, but many other systemic features can impact a retinal microvascular quantitative analysis such as hypertension, hypercholesterolemia, systemic neurovascular, or cardiovascular disease [31,32,33]. Third, the general health characteristics of the control group were self-reported. The systemic clinical examination and blood samples could have been more informative to help to avoid a potential bias. Fourth, our cross-sectional study prevented us from drawing definitive conclusions on the causative or prognostic role of these changes. Further longitudinal follow-up studies investigating retinal vascular densities, diabetic retinopathy, and systemic diabetes characteristics are needed. Moreover, a longitudinal study could also assess whether these subclinical microvascular changes are reversible. Finally, we could not evaluate the link between retinal vascular density modifications and the incidence of clinical diabetic retinopathy.

## 5. Conclusions

Retinal microvascularization was altered in patients with type 1 diabetes without clinical signs of diabetic retinopathy, but intensive insulin treatment and optimized diabetes management with continuous glucose-monitoring devices seemed to attenuate the risk of retinal vascular density rarefaction. Further studies are needed to evaluate the potential clinical impact of these findings.

## Figures and Tables

**Table 1:** Characteristics of the study population.

	Diabetes Group (*n* = 98)		Control Group (*n* = 71)		*p*-Value
		*n*		*n*	
**Laterality (right)**	53 (54%)	98	43 (61%)	71	0.40
**Age (years)**	31.8 (25.3–42.6)	98	29.4 (27.0–35.5)	71	0.36
**Sex (male)**	42 (43%)		28 (39%)		0.66
**Duration of diabetes (years)**	18.0 (8.75–24.0)	96			
**BMI (kg/m^2^)**	24.1 (21.2–27.3)	95			
**Creatinine (μmol/L)**	64.5 (56.0–71.6)	90			
**HbA1c (%)**	7.7 (7.2–8.8)	94			
**MAGE (mg/dL)**	79.5 (63.9–101.0)	46			

BMI: body mass index; HbA1c: glycated hemoglobin; MAGE: mean amplitude glycemic excursion. Descriptive results are presented as *n* (%) and median (interquartile range). Pearson’s chi-squared test was used to compare categorical variables and the Mann–Whitney test was used to compare the quantitative data.

**Table 2:** Comparison of retinal microvascularization between diabetes and control groups.

	Diabetic Group	Control Group	Multivariate Analysis (*n* = 169)	
	Median (Q25–75)	Median (Q25–75)	β (95% CI)	*p*-Value
**FAZ**				
Size (mm^2^)	0.223 (0.167–0.310)	0.221 (0.173–0.294)	−0.115 (−0.818; 0.0353)	0.62
Circularity index	0.730 (0.657–0.769)	0.762 (0.680–0.801)	0.0231 (−0.00559; 0.0572)	0.16
**SCP**				
Central density (mm^−1^)	11.4 (8.82–13.0)	12.3 (10.3–13.8)	0.677 (−0.298; 1.65)	0.17
Inner circle density (mm^−1^)	17.6 (16.1–18.8)	18.4 (17.2–19.2)	1.00 (0.468; 1.79)	<0.01 *
Inner superior density (mm^−1^)	18.5 (16.9–19.6)	19.4 (18.3–20.1)	0.981 (0.475; 1.61)	<0.01 *
Inner nasal density (mm^−1^)	18.6 (17.2–19.8)	19.3 (18.3–20.0)	1.08 (0.502; 1.86)	**<0.01**
Inner inferior density (mm^−1^)	18.4 (17.1–19.5)	19.3 (17.8–20.1)	1.06 (0.469; 1.83)	<0.01 *
Inner temporal density (mm^−1^)	18.3 (17.1–19.6)	18.6 (17.9–19.9)	0.912 (0.136; 1.69)	**0.021**
Outer circle density (mm^−1^)	18.6 (17.4–19.4)	19.0 (18.1–19.6)	0.509 (0.107; 1.08)	0.086
**DCP**				
Central density (mm^−1^)	0.633 (0.264–1.34)	0.704 (0.342–1.57)	0.187 (−0.197; 0.761)	0.41
Inner circle density (mm^−1^)	12.3 (9.60–14.0)	12.6 (10.4–14.1)	0.558 (−0.350; 1.53)	0.27
Outer circle density (mm^−1^)	13.4 (10.9–15.1)	13.5 (10.8–15.4)	0.343 (−0.703; 1.39)	0.52 *

FAZ: foveal avascular zone; SCP: superficial capillary plexus; DCP: deep capillary plexus; β: regression coefficient; CI: confidence interval. Multivariate linear regression model adjusted for age and sex. * Age was a confounding factor. Significant *p*-values are highlighted in bold.

**Table 3:** Association between retinal vascular parameters and diabetes characteristics.

	Diabetes Duration (*n* = 96)		HbA1c (*n* = 94)		MAGE (*n* = 46)		Total Daily Insulin (*n* = 64)	
	*r* (95% CI)	*p*-Value	*r* (95% CI)	*p*-Value	*r* (95% CI)	*p*-Value	*r* (95% CI)	*p*-Value
**FAZ**								
Size (mm^2^)	0.000875 (−0.208; 0.209)	0.99	0.0252 (−0.187; 0.235)	0.81	−0.0487 (−0.342; 0.253)	0.75	−0.164 (−0.404; 0.0966) 0.1145	0.20
Circularity index	−0.176 (−0.370; 0.0340)	0.090	−0.0710 (−0.278; 0.142)	0.50	−0.106 (−0.392; 0.199)	0.49	(−0.147; 0.361)	0.38
**SCP**								
Central density (mm^−1^)	−0.0490 (−0.253; 0.159)	0.64	−0.114 (−0.315; 0.0967)	0.27	−0.0998 (−0.387; 0.205)	0.51	0.173 (−0.0833; 0.408)	0.17
Inner circle density (mm^−1^)	−0.0549 (−0.258; 0.153)	0.59	−0.0326 (−0.239; 0.177)	0.76	−0.0371 (−0.332; 0.264)	0.81	0.2063 (−0.049; 0.436)	0.10
Outer circle density (mm^−1^)	−0.0372 (−0.242; 0.170)	0.72	−0.0443 (−0.250; 0.166)	0.67	−0.0139 (−0.311; 0.286)	0.93	0.162 (−0.095; 0.398)	0.20
**DCP**								
Central density (mm^−1^)	−0.176 (−0.394; 0.0596)	0.13	−0.028 (−0.263; 0.210)	0.82	−0.106 (−0.424; 0.235)	0.53	0.294 (0.00223; 0.539)	**0.042**
Inner circle density (mm^−1^)	−0.0699 (−0.272; 0.138)	0.49	0.0218 (−0.187; 0.229)	0.84	−0.173 (−0.448; 0.132)	0.25	0.08217 (−0.174; 0.328)	0.52
Outer circle density (mm^−1^)	−0.0126 (−0.218; 0.194)	0.90	−0.0596 (−0.265; 0.151)	0.57	−0.132 (−0.414; 0.173)	0.39	0.009891 (−0.244; 0.262)	0.94

FAZ: foveal avascular zone; SCP: superficial capillary plexus; DCP: deep capillary plexus; HbA1c: glycated hemoglobin; MAGE: mean amplitude glycemic excursion; r: Spearman correlation coefficient; CI: confidence interval. Non-parametric Spearman correlation was used to compare quantitative data. Significant *p*-values are highlighted in bold.

**Table 4:** Association between retinal vascular parameters and the use of an FSL device.

	Equipped with FSL Device (*n* = 46)	No FSL Device (*n* = 52)	Multivariate Analysis	
	Median (Q25–75)	Median (Q25–75)	β (95% CI)	*p*-Value
**FAZ**				
Size (mm^2^)	0.210 (0.165–0.299)	0.250 (0.176–0.310)	−0.240 (−1.36; 0.0189)	0.31
Circularity index	0.728 (0.672–0.777)	0.730 (0.650–0.765)	0.00353 (−0.0369; 0.0467)	0.87
**SCP**				
Central density (mm^−1^)	12.3 (9.21–13.3)	11.0 (8.59–12.5)	0.805 (−0.498; 2.11)	0.22 *
Inner circle density (mm^−1^)	18.4 (16.1–19.1)	17.4 (16.2–18.2)	0.871 (−0.0489; 1.93)	0.11 *
Outer circle density (mm^−1^)	19.1 (17.9–19.8)	18.2 (17.2–18.9)	0.922 (0.226; 1.87)	**0.034**
**DCP**				
Central density (mm^−1^)	0.812 (0.358–1.82)	0.432 (0.209–1.14)	0.595 (0.103; 1.45)	0.052
Inner circle density (mm^−1^)	13.8 (11.7–14.8)	11.5 (9.06–12.9)	1.92 (0.520; 3.24)	**<0.01** *
Outer circle density (mm^−1^)	14.3 (12.4–15.7)	12.9 (9.95–14.0)	1.80 (0.427; 3.24)	**0.023**

FAZ: foveal avascular zone; SCP: superficial capillary plexus; DCP: deep capillary plexus; FSL: FreeStyle Libre; β: regression coefficient; CI: confidence interval. Multivariate linear regression model adjusted for age and sex. * Age was a confounding factor. Significant *p*-values are highlighted in bold.

## Data Availability

Presented data are available on request from the corresponding author.
